# Synthesis, Bioactivity, Molecular Docking and POM Analyses of Novel Substituted Thieno[2,3-*b*]thiophenes and Related Congeners

**DOI:** 10.3390/molecules20021824

**Published:** 2015-01-23

**Authors:** Yahia N. Mabkhot, Fahad D. Aldawsari, Salim S. Al-Showiman, Assem Barakat, Taibi Ben Hadda, Mohammad S. Mubarak, Sehrish Naz, Zaheer Ul-Haq, Abdur Rauf

**Affiliations:** 1 Department of Chemistry, College of Science, King Saud University, P.O. Box 2455, Riyadh 11451, Saudi Arabia; E-Mail: showiman@ksu.edu.sa; 2 King Abdulaziz City for Science and Technology, P.O. Box 6086, Riyadh 11442, Saudi Arabia; E-Mail: fzsw2010@hotmail.com; 3 Department of Chemistry, Faculty of Science, Alexandria University, P.O. Box 426, Ibrahimia 21321 Alexandria, Egypt; 4 Lab of Chemical Material, Faculty of Sciences University Mohammed Premier, Oujda 60000, Morocco; E-Mail: taibi.ben.hadda@gmail.com; 5 Department of Chemistry, The University of Jordan, Amman 11942, Jordan; E-Mail: mmubarak700@gmail.com; 6 Dr. Panjwani Center for Molecular Medicine and Drug Research, International Center for Chemical and Biological Sciences, University of Karachi, Karachi 75210, Pakistan; E-Mails: mjazse@gmail.com (S.N.); zaheer.qasmi@iccs.edu (Z.U.-H.); 7 Institute of Chemical Sciences, University of Peshawar, Peshawar 25120, Pakistan; E-Mail: mashaljcs@yahoo.com

**Keywords:** thienothiophene, antibacterial activity, antifungal activity, molecular docking, Petra/Osiris/Molinspiration (POM) analyses

## Abstract

Several series of novel substituted thienothiophene derivatives were synthesized by reacting the synthone **1** with different reagents. The newly synthesized compounds were characterized by means of different spectroscopic methods such as IR, NMR, mass spectrometry and by elemental analyses. The new compounds displayed significant activity against both Gram-positive and Gram negative bacteria, in addition to fungi. Molecular docking and POM analyses show the crucial role and impact of substituents on bioactivity and indicate the unfavorable structural parameters in actual drug design: more substitution doesn’t guaranty more efficiency in bioactivity.

## 1. Introduction

The thieno[2,3-*b*]thiophene moiety has been used in the design of a novel non-linear optical (NLO) properties system by incorporating this nucleus within an unsymmetrically functionalized cyclophane; this was first described by Mashraqui *et al.* [1,2]. Additionally, thienothiophene building blocks have been used for the preparation of two series of human immunodeficiency virus (HIV) protease inhibitors possessing an α-hydroxyaminopentanamide transition state isoester. At low concentrations, these HIV protease inhibitors were found to be effective at halting the spread of the acquired immunodeficiency syndrome (AIDS) virus within the body [3]. Egbertson *et al.* [4] reported on the synthesis and pharmacology of a potent thienothiophene non-peptide fibrinogen receptor antagonist. In 2003, Sasaki *et al.* [5] described the first potent and orally effective non-peptide antagonist for the human luteinizing hormone-releasing hormone receptor.

Due to their structural and therapeutic diversity, thienothiophene derivatives have attracted much synthetic interest because of their reactivity and biological activity, and have drawn considerable attention from researchers. They have been examined as potential antitumor, antiviral, antibacterial, anti-glaucoma drugs, and as inhibitors of platelet aggregation [6,7,8,9,10,11,12,13,14,15,16,17,18].

In 2010, Paek and coworkers have designed and synthesized six organic sensitizers containing 3,4-ethylenedioxythiophene and thienothiophene; these compounds have shown high efficiency and excellent stability [19]. Recently, Mabkhot *et al.* have reported for the first time on the anti-oxidant, *β*-glucuronidase and *α*-glucosidase inhibition potential of thieno[2,3-*b*]thiophene derivatives [20].

On the other hand, addition of the enaminone group to the thienothiophene nucleus has added new activities to these compounds; one derivative that contains both of these groups is used as a drug to treat epilepsy [21,22]. In view of the wide interest in the activity and profile of thienothiophenes and enaminones, and in the search for new therapeutic agents, we describe herein the synthesis and characterization of a number of new substituted thieno[2,3-*b*]thiophenes which, to the best of our knowledge, have not previously been described in the literature. In addition, the bioactivity of the newly prepared compounds will be presented.

## 2. Results and Discussion

### 2.1. Chemistry

**Scheme 1:**
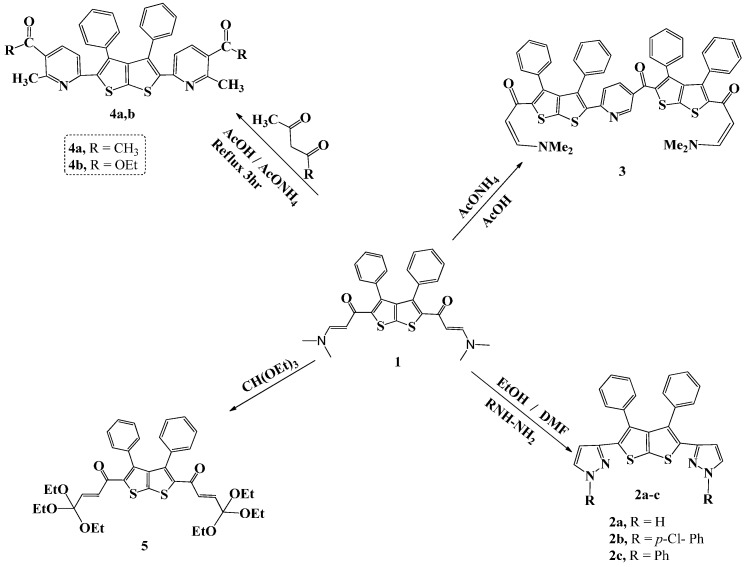
Synthesis of compounds **1**–**5**.

**Scheme 2:**
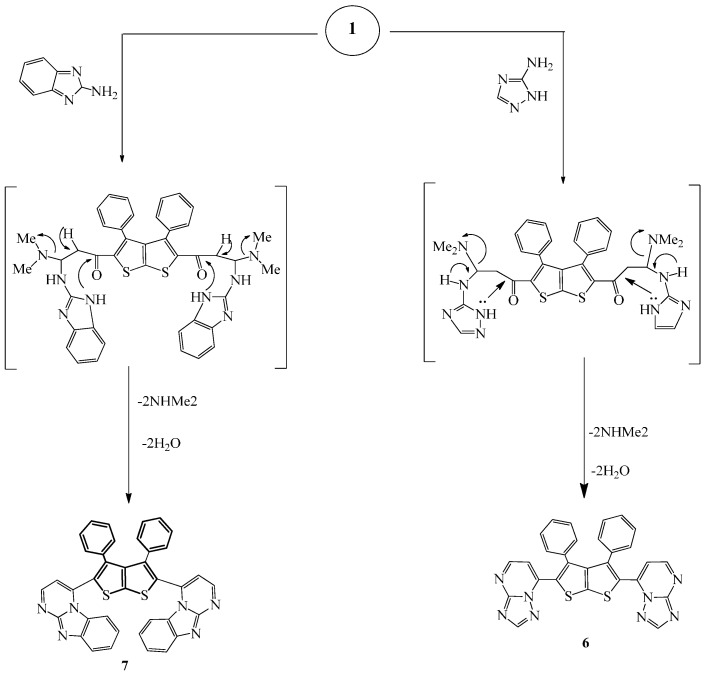
Synthesis of compounds **6** and **7**.

Compounds **1**–**7** were synthesized as shown in Scheme 1 and Scheme 2. The newly synthesized compounds **1**–**7** were characterized by elemental analyses, MS and NMR spectral data. These data, detailed in the experimental part, are consistent with the suggested structures. Thus, the mass spectra display the correct molecular ion peaks for which the measured high resolution (HRMS) data are in good agreement with the calculated values. DEPT and 2D (COSY, HMQC, HMBC) experiments showed correlations that helped in the ^1^H- and ^13^C-signal assignments to the different carbons and their attached, and/or neighboring hydrogens.

Compound **1**, a synthon required in this study, was prepared by following a procedure outlined by Mabkhot, *et al.* [23]. Compounds **2a**, **2b**, and **2c** were prepared through the reaction of compound **1** with the appropriate hydrazine in ethanol/DMF as solvent as shown in Scheme 1. Compound **3**, on the other hand, was synthesized by refluxing a mixture of the enaminone **1** with ammonium acetate in acetic acid as solvent for 5 h.

The IR spectrum of compound **1** exhibited an absorption band at 1619 cm^−1^ ascribed to the carbonyl group (C=O). Similarly, reaction of **1** with acetyl acetone and with ethyl acetoacetate in the presence of ammonium acetate in acetic acid as solvent, under reflux for 6 h afforded **4a** and **4b**, respectively. Compound **5** was prepared by fusing compound **1** with triethylorthoformate (TEOF) followed by the addition of ethanol/DMF (10 mL, 1:3) as solvent. The precipitate that resulted was filtered and its structure confirmed by spectroscopic methods. The IR spectrum showed absorption bands at 1626 and 3050 cm^−1^ due to C=O and C-H stretching, respectively. ^1^H-NMR spectrum of compound **5** showed a triplet at δ 1.05, and a quartet at 2.84–3.13 ppm, due to the methyl and CH_2_ of the ether group. In addition, two doublets for the CH=CH protons at 5.69 (*J* = 12.0 Hz) and 6.15 (*J* = 12.0 Hz) were observed in addition to a multiplet at δ 7.42–7.52 attributed to the phenyl protons. ^13^C-NMR spectrum agrees well with the suggested structure and showed the following signals: δ 22.4 (-CH_3_), 44.0 (-OCH_2_), 129.3, 129.9 (Ph), 130.5, 131.5, 133.3, 142.0 (Ar-C), 182.4 (C=O). The mass spectrum displayed the molecular ion [M]^+^ (19%) at *m*/*z* 694 corresponding to the molecular formula C_38_H_46_O_8_S_2_ in addition to fragments at 98 (100%) and 57 (89%).

Compound **6** was prepared by refluxing a mixture of compound **1** and 3-amino-1*H*-1,2,4-triazole in methanol/DMF (Scheme 2). The IR spectrum of the prepared compound showed absorption bands at 1588 and 2924 cm^−1^ due to C=N and C-H, respectively.

Its ^1^H-NMR spectrum recorded in DMSO-*d*_6_ showed a multiplet at δ 7.41–7.66 due to the aromatic hydrogens in addition to two doublets at δ 8.14 (*J* = 12.5 Hz) and 8.96 (*J* = 12.5 Hz) attributed to the two CH protons of the pyrimidine ring and to a singlet at 8.67 of the triazole hydrogen. Moreover, the ^13^C-NMR spectrum of the prepared compound is in agreement with the proposed structure and showed the following signals pertaining to the different carbon atoms: δ 115.4 (CH), 127.0, 129.1, 130.0 (Ph), 125.0, 130.2, 132.8, 135.8 (thiophene), 152.8, 154.4, 160.0, and 162.9 (Ar-C). Further support of the structure came from mass spectral data; mass spectrum of compound **6** displayed the correct molecular ion [M]^+^ at *m*/*z* = 530 corresponding to the molecular formula C_28_H_18_N_8_S_2_. Fragments at *m*/*z* 81 (86%), 67 (100%), and 55 (88%), in addition to other small ones have also appeared. Other prominent fragments at 368 (100%), 165 (98%), and 105 (40%) also appeared.

Finally, compound **7** was prepared by refluxing a mixture of **1** and 2-aminobenzimidazole in EtOH/DMF for 7 h (Scheme 2). The IR spectrum of the prepared compound showed characteristic absorption bands at 1626 (C=N), 1539 cm^−1^ (C=C) in addition to the disappearance of the C=O absorption band of compound **1**. ^1^H-NMR spectrum in DMSO-*d*_6_ displayed two doublets corresponding to the pyrimidine CH protons at δ 8.16 (*J* = 12.5 Hz) and 8.80 (*J* = 12.5 Hz) in addition to a multiplet corresponding to the phenyl and to the benzimidazole rings in the range δ 7.42–7.52. ^13^C-NMR spectrum is also in agreement with the suggested structure; it showed signals corresponding to the different carbon atoms in the compound as follows: δ 112.1, 115.1, 122.5, 127.1, 128.1, 129.9, 131.3 ,136.0, 139.1, 142.0, 148.1, 148.4, 156.0 (Ar-C). Similarly, the mass spectrum showed the correct molecular ion [M]^+^ (25%) at *m*/*z* = 648 corresponding to the molecular formula C_38_H_24_N_6_S_2_ in addition to other related fragments at *m*/*z* = 439 (55%), 183 (100%) and at 57 (64%).

### 2.2. Antimicrobial Activity

To investigate the biological activity of the newly prepared compounds, the Cup-plate agar diffusion method was adopted by using discs of disinfected filter papers (6 mm in diameter). Tested compounds were dissolved in DMSO and were charged on the discs with concentrations of 5 mg/mL. The discs were then placed in Petri dishes and were loaded with different Gram-positive and Gram negative bacterial strains: *Pseudomoas aeruginosa* (RCMB 010043) and *Escherichia coli* (RCMB 010052) for Gram-negative bacteria and *Staphylococcus pneumonia* (RCMB 010010), *Bacillis subtilis* (RCMB 010067) for Gram-positive, and *Aspergillus fumigates* (RCMB 02568) and *Candida albicans* (RCMB 05036) for fungi. Results of the biological activity are displayed in Table 1 as mm inhibition.

**Table 1:** Antibacterial and antifungal activity of synthesized compounds.

Compd.	Fungi ^[a]^		Gram (+) Bacteria ^[b]^		Gram (−) Bacteria ^[c]^	
	*(A)*	*(B)*	*(C)*	*(D)*	*(E)*	*(F)*
**1**	17.3 ± 0.4	16.9 ± 0.3	16.3 ± 0.6	18.3 ± 0.3	NA	NA
**2a**	20.2 ± 06	19.6 ± 0.3	23.8 ± 0.2	32.4 ± 0.3	17.3 ± 0.1	19.9 ± 0.3
**2b**	16.7 ± 0.4	15.8 ± 0.5	10.8 ± 0.4	11.9 ± 0.3	10.8 ± 0.4	9.7 ± 0.5
**3**	15.9 ± 0.4	15.8 ± 0.5	10.8 ± 0.4	11.9 ± 0.3	10.8 ± 0.4	9.7 ± 0.5
**4a**	18.1 ± 0.5	14.6 ± 0.5	17.9 ± 0.5	13.3 ± 0.4	11.4 ± 0.4	10.7 ± 0.3
**4b**	14.6 ± 0.4	15.9 ± 0.5	10.2 ± 0.2	9.8 ± 0.3	NA	11.2 ± 0.3
**5**	13.9 ± 0.4	14.6 ± 0.5	16.3 ± 0.5	19.6 ± 0.6	12.5 ± 0.4	12.8 ± 0.4
**6**	12.8 ± 0.3	15.4 ± 0.5	14.1 ± 0.5	12.7 ± 0.4	11.6 ± 0.4	9.1 ± 0.4
**7**	18.1 ± 0.6	15.9 ± 0.5	12.6 ± 0.5	13.7 ± 0.6	12.1 ± 0.4	10.4 ± 0.2
**SD-1 ^[d]^**	23.7 ± 0.1	25.4 ± 0.1	23.8 ± 0.2	32.4 ± 0.3	---	---
**SD-2 ^[e]^**	---	---	---	---	17.3 ± 0.1	19.9 ± 0.3

Notes: ^[a]^ *(A):* *Aspergillus fumigatus*, *(B):* *Candida albicans*; ^[b]^ *(C):* *Staphylococcus aureus*, *(D): Bacilils subtilis*; ^[c]^ *(E)*: *Pseudomonas aeruginosa*, *(F): Escherichia coli*; ^[d]^ **SD-1**: Streptomycin for antimicrobial (25 µg/mL); ^[e]^ **SD-2**: Clotrimazole standered drug for fungi (25 µg/mL).

Results shown in Table 1 reveal that compound **2a** has exhibited remarkable activity against both Gram-positive and negative bacteria while compounds **4a**, **5** and **6** showed good activity against Gram-positive bacteria. On the other hand, compound **2a** was the most active against tested fungus. Results also reveal that compound **2a** was the most potent; it has antibacterial and antifungal activities due to the presence of electron-rich functional groups. These groups could bind with the microorganisms. In addition, the presence of acrylides bonded to the thionothiophene nucleus and the effect of thionothiophene itself against bacteria and fungi may explain the potency of these compounds; the tautomeric forms of these compounds penetrate the cell wall.

### 2.3. Molecular Docking Studies

Molecular docking is a technique which serves to verify binding integrity and interaction poses of ligands within the binding pocket of target proteins [24]. To validate and specify the target for anti-fungal and anti-bacterial activity of newly synthesized thieno[2,3-*b*]thiophene derivatives, nine different target proteins *i.e.*, dihydrofolate reductase (DHFR) (PDB ID 4HOF), secreted aspartic protease (PDB ID 3Q70), and N-myristoyl transferase (PDB ID 1IYL) from *C. albicans* were selected as fungal targets, while for bacterial targets dihydrofolate reductase (PDB ID 3FYV), gyrase B (PDB ID 4URM) and sortase A (PDB ID 2MLM) from *S. aureus* and rhomboid protease (PDB ID 3ZMI), methionine peptidase (PDB ID 4PNC) and undecaprenyl diphosphate synthase (PDB ID 4H2M) from *E. coli* were fetched from the Protein Data Bank [25]. Among all these nine target proteins, only two target proteins, *i.e.*, dihydrofolate reductase (DHFR) from *C. albicans* and rhomboid protease from *E. coli* were preferred as they showed good interactions and binding affinities with the synthesized compounds in docking simulation by MOE 2013 [26], while the others have binding pockets that are too small to fit these large compounds. Before docking, structures of synthesized compounds were built and saved in their 3D conformation by MOE 2013. Moreover, for target protein preparation, all nine proteins were prepared, charged, protonated and minimized by MOE 2013. The 4HOF is a homotrimer (ABC chains) and 3ZMI is a monomer with bound inhibitors 18H and L6C, respectively. Chain A of 4HOF and a single chain of 3ZMI with one conserved water molecule (HOH2016) were selected for the evaluation of newly synthesized compounds. Both of these proteins were prepared, charged, protonated and minimized by MOE 2013. For docking, default MOE docking parameters *i.e.*, Triangle Matcher Algorithm with two rescoring functions London dG and GBVI/WSA dG were utilized to generate 30 poses of each compound. As a result, mdb output files were generated enclosing all docking results with scoring and multiple conformations of ligands. Results were finally inspected to determine the most potent and effective anti-fungal and anti-bacterial inhibitor by visualizing various interactions of ligands within binding pocket.

**Figure 1:**
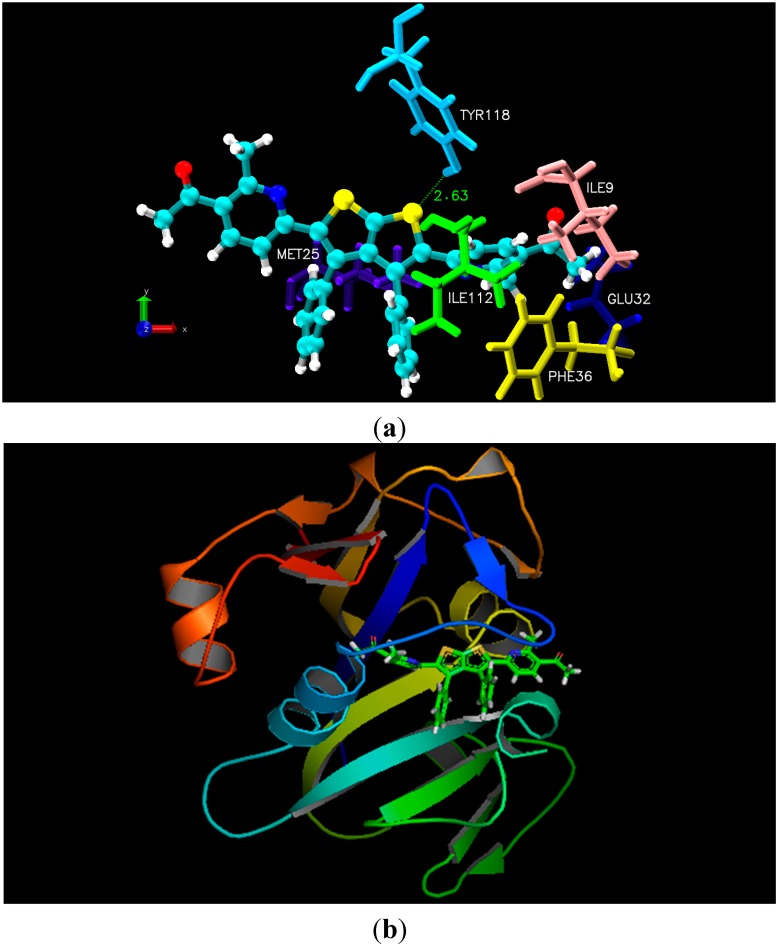
(**a**) Molecular docking interaction model for the most potent anti-fungal compound **4a** with *C. albicans* dihydrofolate reductase protein (PDB ID.4HOF) showing hydrogen bonding, hydrophobic and Van der Waals interactions; (**b**) ribbon diagram of dihydrofolate reductase (*C. albicans*) complex with inhibitor **4a**.

**Figure 2:**
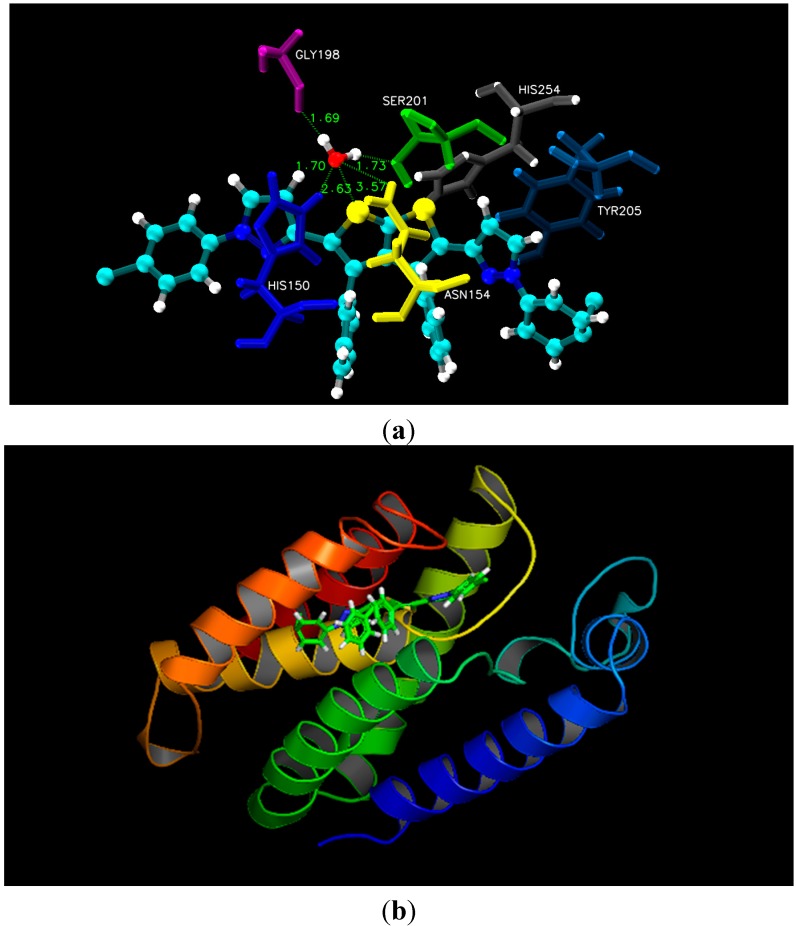
(**a**) Molecular docking interaction model for the most potent anti-bacterial compound **2b** with rhomboid protease of *E. coli* (PDB ID.3ZMI) showing hydrogen bonding through conserved water molecule & hydrophobic interactions; (**b**) ribbon diagram of rhomboid protease (*E. coli*) complex with inhibitor **2b**.

As illustrated in the experimental results, docking studies of all nine thieno[2,3-*b*]thiophene derivatives unveiled their potency as anti-fungal and anti-bacterial inhibitors. All newly synthesized compounds contain large aromatic systems, exhibiting noticeable hydrophobic and Van der Waals (VDW) interactions along with few additional hydrogen bonding with the crucial residues *i.e.*, Ile9, Glu32, Phe36, Ile112, and Tyr118 of 4HOF (*C. albicans*) [27]. However, 3ZMI (*E. coli*) His150, Asn154, Ser201, and His254 were observed to be the hotspot residues for their inhibitory activity [28]. Docking results indicated that all newly synthesized compounds, especially **4a** and **2b**, displayed pronounced hydrophobic and Van der Waals interactions due to the presence of electronegative functionalities. Unfortunately, compounds **3**, **5** and **6** being most potent inhibitors like **4a** and **2b**, did not show better interactions due to lack of fit in the cavity of the target proteins, due to the presence of bulky R-group that create steric hindrance whereas few compounds lack certain electronegative functionalities at R-group. Moreover, docking results with 4HOF presented hydrogen bonding of the thiol ring sulphur (compound **4a**) with the hydroxyl group of Tyr118 at a distance of 2.63 Å, whereas the pyrimidine ring of **4a** displayed hydrophobic interactions with the aromatic ring of Phe36. In addition, various Van der Waals interactions were observed with other prime residues *i.e.*, Glu32, Ile9 and Ile112 (Figure 1). Alternatively, docking simulation with 3ZMI revealed that sulphur of the thiol ring of compound **2b** was involved in hydrogen bonding with His150, Asn154 and Ser201, where conserved water molecule (HOH2016) acts as a bridging atom. Additionally, both pyrol rings of compound **2b** displayed hydrophobic interaction with Tyr205 and His150 (Figure 2). According to our docking simulation studies by MOE 2013, we concluded that all thieno[2,3-*b*]thiophene derivatives, especially compounds **4a** and **2b**, exhibited marked hydrogen bonding, hydrophobic and Van der Waals interactions with vital residues of DHFR of *C. albicans* and rhomboid protease of *E. coli* and are the most active anti-fungal and anti-bacterial inhibitors, respectively.

### 2.4. POM Analyses of Compounds **1**–**7**

POM theory is able to identify the type of pharmacophoric sites [29]. POM has certainly become one of the best-known recent methods that are regularly used [30] to produce two dimensional models to identify and to indicate the type of pharmacophoric sites that affect antimicrobial, antiviral, and antifungal activities with a change in the chemical substitution and partial charge distribution. In fact, the real advantages of POM theory are the ability to easily predict the biological activities of molecules and to show the relationship between steric/electrostatic properties and biological activity in the form of pharmacophoric sites; this gives key features not only on the ligand-receptor interaction, but also on the topology of the receptor with the coexistence of tautomerism, isomerism, and ring opening/closing processes [31].

For a molecule to be a potential drug, besides having a good biological activity, it must have good pharmacokinetic properties in biological systems. To access the pharmacokinetic profile of synthesized molecules, we employed well established *in silico* tools such as Osiris, Petra and Molinspiration validated with about 7000 drug molecules available on the market and created new economic and effecient bioinformatic platform called POM Analyses [31]. Now POM theory is well verified and it is more and more popular by many other research groups involved in bioinformatics and drug design.

One of the practical problems associated with synthetic drugs is the existence of various side effects. Shown in Table 2 are results of theoretical toxicity risks of compounds **1**–**7** calculated with the aid of the Petra/Osiris/molinspiration (POM) program [32,33,34,35]. Our findings reveal that compounds **2**, **4**, **6** and **7**, contrary to compounds **1**, **3** and **5**, are not toxic and can be utilized as therapeutic agents. In addition, results presented in Table 2 show that structures of the investigated compounds are supposed to be non-mutagenic when run through the mutagenicity assessment of free system, and that these compounds are at low risk comparable with standard synthetic drugs as far as irritation and reproductive effects are concerned.

**Table 2:** Osiris calculations of toxicity risks of compounds **1**–**7**.

**Compd.**	**MW**	**Toxicity Risks ^[a]^**				**Osiris Calculations ^[b]^**			
		MUT	TUM	IRRIT	REP	cLog*P*	Sol	DL	DS
**1**	486	+++	+++	++	++	3.89	−8.81	3.61	0.22
**2a**	424	+++	+++	+++	++	4.75	−8.72	3.15	0.27
**2b**	644	+++	+++	+++	++	8.51	−12.52	4.66	0.12
**2c**	576	+++	+++	+++	++	7.31	11.01	4.51	0.14
**3**	881	+++	+++	++	++	9.41	−18.31	3.61	0.08
**4a**	558	+++	+++	+++	++	7.51	−12.02	1.37	0.13
**4b**	784	+++	+++	+++	++	8.39	−11.60	−2.32	0.07
**5**	692	+++	+++	++	++	7.97	−10.71	−15.35	0.05
**6**	528	+++	+++	+++	++	4.16	−7.01	5.79	0.27
**7**	626	+++	+++	+++	++	8.24	−11.54	4.63	0.12
**SD-1 ^[c]^**	581	+++	+++	---	+++	7.86	−0.96	0.83	0.32
**SD-2 ^[d]^**	344	+++	+++	+++	+++	5.37	−7.72	0.92	0.30

Notes: Higly toxic: (---), Slightly toxic: (+), Not toxic (+++); ^[a]^ MUT: Mutagenic, TUM: Tumorigenic, IRRIT: Irritant, REP: Reproductive effective; ^[b]^ Sol: Solubility, DL: Drug likeness; ^[c]^ **SD-1**: Streptomycin for antimicrobial (25 µg/mL); ^[d]^ **SD-2**: Clotrimazole standered drug for fungi (25 µg/mL).

The hydrophilicity character of each compound has been expressed in terms its cLog*P* value since it has been established that the absorption or permeation is greatly affected by this quantity (value of cLog*P*). Accordingly, when the value of cLog*P* is higher than 5, the absorption or permeation decreases. Our results show that only 3/10 compounds (**1**, **2a** and **6**) have cLog*P* values within the acceptable criteria and are potentially active against various biotargets (GPCRL: GPCR ligand; ICM: Ion channel modulator; KI: Kinase inhibitor; NRL: Nuclear receptor ligand; PI: Protease inhibitor; EI: Enzyme inhibitor) as shown in Table 2. Thus, the cLog*P* parameter should be taken into consideration and serve as a guide for further enzymatic screening investigations.

Similarly, the geometrical conformation of pharmacophores in this context is important since it is not fixed for compounds **1**–**7**. Properties such as absorption, distribution characteristics, and bioactivity depend on the geometrical parameter and the aqueous solubility of each compound. Consequently, good absorption of tested compounds **1**–**7** could presumably be due to their good solubility [34,35]. Furthermore, Table 3 shows drug-likeness of compounds **1**–**7**, in the incomparable zone with standard known drugs (**SD-1**: streptomycin for antimicrobial and **SD-2**: clotrimazole as standard drug for fungi). For example, we have calculated an overall drug-score (DS) for compound **1** and compared it with that of compound **2a** and concluded that **2a** was better than rest of series **1**–**7**, except **6**). The DS combines drug-likeness, cLog*P*, log*S*, molecular weight, and toxicity risks, in a one handy value that may be used to judge the compound’s overall potential to qualify for a drug. As shown in Table 3, the reported compounds **1**–**7** showed low to moderate DS (DS < 0.50).

**Table 3:** Molinspirationcalculations of compounds (**1**–**7**).

**Compd.**	**Molinspiration Calculations ^[a]^**				**Drug-Likeness ^[b]^**					
	TPSA	NONH	NV	VOL	GPCRL	ICM	KI	NRL	PI	EI
**1**	41	0	1	437	−0.04	−0.34	−0.21	−0.35	−0.06	0.10
**2a**	57	2	1	357	0.09	−0.02	0.12	−0.28	0.01	0.13
**2b**	36	0	2	527	−0.08	−0.64	−0.38	−0.51	−0.10	−0.29
**2c**	36	0	2	500	0.01	−0.43	−0.22	−0.34	−0.04	−0.13
**3**	91	1	2	754	−2.72	−3.66	−3.32	−3.57	−2.11	−2.92
**4a**	60	0	2	491	0.08	−0.35	−0.10	−0.20	−0.03	−0.04
**4b**	78	0	2	543	−0.16	−0.72	−0.32	−0.43	−0.11	−0.27
**5**	90	0	2	632	−0.47	−1.28	−0.91	−0.96	−0.23	−0.64
**6**	86	0	2	429	−0.01	−0.37	0.07	−0.53	−0.10	−0.05
**7**	60	0	2	525	−0.19	−0.82	−0.38	−0.87	−0.24	−0.50
**SD-1 ^[c]^**	336	16	3	497	0.09	−0.16	−0.17	−0.18	0.65	0.38
**SD-2 ^[d]^**	18	0	1	310	0.17	0.30	0.14	−0.21	−0.13	0.42

Notes: ^[a]^ TPSA: Total molecular polar surface area; NONH: number of OH---N or O---NH interaction, NV: number of violation of five Lipinsky rules; VOL: volume. ^[b]^ GPCRL: GPCR ligand; ICM: Ion channel modulator; KI: Kinase inhibitor; NRL: Nuclear receptor ligand; PI: Protease inhibitor; EI: Enzyme inhibitor. ^[c]^ **SD-1**: Streptomycin for antimicrobial. ^[d]^ **SD-2**: Clotrimazole standered drug for fungi.

Most importantly, the molinspiration calculations pertaining to compounds **1**–**7** show that most of them, could be good candidates to interact with various enzymatic targets (GPCR ligand, ion channel modulator, kinase inhibitor, nuclear receptor ligand, protease inhibitor and enzyme inhibitor) when the two central phenyl substituents are retrieved from principal skeleton (two methyl instead the two phenyl will be more interesting).

**Figure 3:**
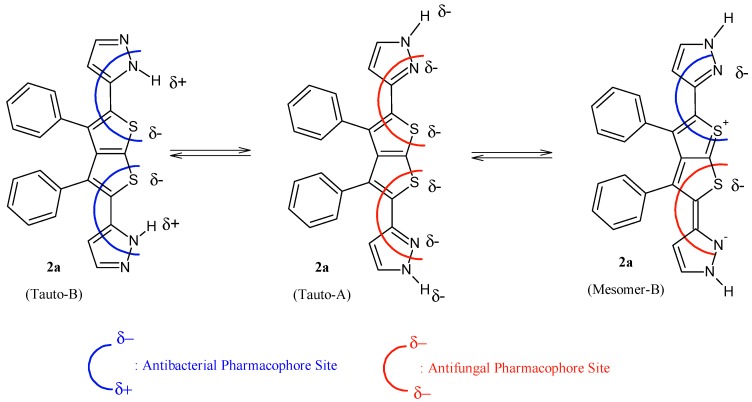
Identification of explicit and combined antibacterial/antifungal pharmacophoric sites of compound **2a**.

**Figure 4:**
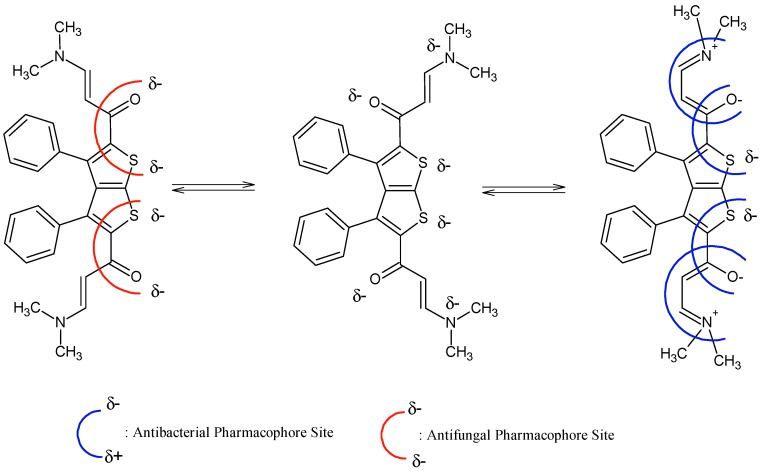
Identification of steric hindered antibacterial and antifungal pharmacophoric sites of compound **1**.

It is evident that compounds **1**–**7** are subjected to important chemical processes of tautomerism/mesomerism, leading to regeneration of combined pharmacophoric sites as shown in Figure 3 and Figure 4 for compounds **1** and **2a**, respectively.

In addition, results from Figure 3 and Figure 4 reveal that compound **2a** has more chances to inhibit microorganisms because its pharmacophoric sites are more explicit than those of the polysubstituted analogue compound **1**. POM analyses thus show the crucial role and impact of substituents on bioactivity and indicate the unfavorable structural parameters in actual drug design: more substitution doesn’t guarantee more efficiency in bioactivity.

## 3. Experimental Section

### 3.1. Materials and Equipment

Chemicals and materials used in this investigation were obtained from commercial sources and were used without further purification. Preparative TLC was performed on Merck Art. 5745, Kieselgel 60 F254/0.50 nm (Merck KGaA, 64271 Darmstadt, Germany). Melting points (uncorrected) were measured with a Electrothermal melting point apparatus (Bibby Scientific Limited, Beacon Road, Stone, Staffordshire, UK). Infrared spectra (IR) were recorded, as potassium bromide (KBr) discs, on a Nicolet 6700 FT-IR spectrophotometer (Madison, WI, USA). ^1^H- (400 MHz) and ^13^C-NMR (100 MHz) spectra were acquired with the aid of a JEOL-400 MHz spectrometer (Jeol, Tokyo, Japan) with DMSO-*d*_6_ as solvents and TMS as an internal standard. Chemical shifts are expressed in δ units; coupling constants (*J*-values) for ^1^H-^1^H are given in Hertz (Jeol, Tokyo, Japan). Mass spectra were recorded on a Jeol JMS-600 H instrument (Tokyo, Japan) at 70 eV. Elemental analyses were performed on a Euro Vector Elemental Analyzer (EA 3000 A, Via Tortona, Milan, Italy).

### 3.2. Preparation of 1,1'-(3,4-Diphenylthieno[2,3-b]thiene-2,5-diyl)bis(3-dimethyl-aminoprop-2-en-1-one) *(***1***)*

A mixture of 1,1'-(3,4-diphenylthieno[2,3-*b*]thiophene-2,5-diyl)diethanone (1.89 g, 5 mmol), DMF-DMA (1.19 mL, 0.01 mol) was refluxed in *m*-xylene (15 mL) for 7 h. After cooling, the resulting solid product was collected by filtration and recrystallized by using DMF/EtOH to give the desired product **1**. Yield: 73%; m.p. 250 °C; IR (_νmax_): 1622 (C=O), 1539, 1457 cm^−1^; ^1^H-NMR (DMSO-*d*_6_) δ (ppm): 2.99 (s, 12H, CH_3_), 4.53 (d, 1H, *J* = 12 Hz, CH), 5.38 (d, 1H, *J* = 12 Hz, CH), 7.41–7.65 (m, 5H, C_6_H_5_); ^13^C-NMR (DMSO-*d*_6_) δ (ppm): 192.2, 153.2, 147.7, 145.8, 141.8, 138.8, 134.8, 129.8, 129.6, 129.2, 125.3, 30.6; MS *m*/*z* (%): 488 [M+, 30%], 386 (100), 368 (47), 213 (73), 43 (46); Anal. calcd. for C_28_H_26_N_2_O_2_S_2_: C, 69.11; H, 5.39; N, 5.76; Found: C, 69.15; H, 5.41; N, 5.78.

### 3.3. General Procedure for the Preparation of Compounds **2a**–**c**

Compounds **2a**–**c** were prepared using the following general procedure: compound **1** (0.244 g, 5.0 mmol) was added to hydrazine hydrate (2 mL), phenylhydrazine (1 mL), or 4-chlorophenyl hydrazine (0.5 g) (to make compounds **2a**–**c**, respectively). The mixture of compound **1** and the appropriate hydrazine was refluxed until compound **1** is completely dissolved, followed by addition of the mixed solvent EtOH/DMF. The reaction mixture was then refluxed for 6–7 h and the product was filtered after cooling. The desired product was obtained in a pure form by recrystallization from EtOH/DMF. Using the general procedure, the following compounds were prepared.

#### 3.3.1*.* 3,3'-(3,4-Diphenylthieno[2,3-*b*]thiophene-2,5-diyl)bis(1*H*-pyrazole) (**2a**)

Yellow crystals; yield: 89%; mp: 133–135 °C. IR (cm^−1^): 3192 (NH); 1508 (C=N). ^1^H-NMR (DMSO-*d*_6_) δ (ppm): 6.45 (d, *J* = 4.5 Hz, CH), 7.40–7.53 (m, 5H), 7.81 (d, *J* = 4.5 Hz, CH), 13.00 (s, 1H, NH). ^13^C-NMR δ: 103.0 (CH), 127.3 (CH), 128.6 (CH), 129.0 (C), 129.1 (CH), 130.0 (C), 130.4 (C), 131 (N-CH), 131.3 (C), 135.0 (C), 136.4 (C), 145.8 (C), 147.3 (Ar-C). MS *m*/*z* (70 eV): 580, M^+^ (4%); 279, [M−301]^+^ (100%); 189, [M−391]^+^ (46%); 104, [M−476]^+^ (33%). Anal. Calcd for C_24_H_16_N_4_S_2_: C, 67.90; H, 3.80; N, 13.20; S, 15.10. Found: C, 67.78; H, 4.21; N, 13.00; S, 15.19.

#### 3.3.2. 3,3'-(3,4-Diphenylthieno[2,3-*b*]thiophene-2,5-diyl)bis(1-chloro phenyl-1*H*-pyrazole) (**2b**)

Red powder; yield: 67%; mp: 339–341 °C. IR (cm^−1^): 1610. ^1^H-NMR (DMSO-*d*_6_): δ, 6.48 (d, *J* = 4.5 Hz, CH), 6.53–7.20 (m, 9H, C_6_H_5_), 7.55 (d, *J* = 4.5 Hz, 1H). ^13^C-NMR: δ, 102.0 (CH), 127.3 (CH), 128.5 (CH), 132.0 (N-CH), 129.2 (CH), 129.9 (C), 130.4 (C), 130.5, 136.4 (C), 145.8 (C), 147.3 (C), 152.8 (C). MS *m*/*z* (70 eV): 646, [M−301]^+^ (100%); 189, [M−391]^+^ (46%); 104, [M−476]^+^ (33%). Anal. Calcd for C_36_H_22_Cl_2_N_4_S_2_: C, C, 66.97; H, 3.43; Cl, 10.98; N, 8.68; S, 9.93. Found: C, 66.99; H, 3.47; Cl, 11.00; N, 8.55; S, 9.78.

#### 3.3.3. 3,3'-(3,4-Diphenylthieno[2,3-*b*]thiophene-2,5-diyl)bis(1-phenyl-1*H*-pyrazole) (**2c**)

Brown powder; yield: 64%; mp: 194–196 °C. IR (cm^−1^): 1618. ^1^H-NMR (DMSO-*d*_6_): δ, 6.50 (d, *J* = 4.5 Hz, 1H), 6.53–7.20 (m, 9H, C_6_H_5_), 7.57 (d, *J* = 4.5 Hz, 1H). ^13^C-NMR: δ, 102.1 (CH), 132.0 (N-CH), 128.4, 190.4, 130.5, 131.5, 132.0, 132.9, 133.50, 141.4, 153.3, 160.8 (Ar-C). MS *m*/*z* (70 eV): 576, M^+^ (694%); Anal. Calcd for C_36_H_24_N_4_S_2_ C, 74.97; H, 4.19; N, 9.71; S, 11.12. Found: C, 74.87; H, 4.00; N, 9.83; S, 10.92.

### 3.4. Synthesis of 3-(Dimethylamino)-1-(5-(6-(5-((E)-3-(dimethylamino)acryloyl)-3,4-diphenylthieno[2,3-b]thiophen-2-yl)nicotinoyl)-3-methyl-4-phenylthieno[2,3-b]thiophen-2-yl)prop-2-en-1-one *(***3***)*

Compound **1** (0.244 g, 0.5 mmol) was dissolved in glacial acetic acid (10 mL) containing ammonium acetate (1.0 g) in a 100-mL round bottomed flask connected to a condenser. The mixture was refluxed for 5 h and after cooling, the precipitate was filtered and recrystallized from DMF/EtOH to afford the desired product as yellow powder. Yield: 75%; mp: 298–300 °C. IR (cm^−1^): 1618. The product was insoluble in most organic solvents, which prevented us from recording its ^1^H- and ^13^C-NMR spectra. MS *m*/*z* (70 eV): 821, M^+^ (2%); 262, [M−559]^+^ (27%); 67, [M−754]^+^ (100%). Anal. Calcd for C_52_H_39_N_3_O_3_S_4_: C, 70.80; H, 4.46; N, 4.76; S, 14.54; Found: C, 70.81; H, 4.46; N, 4.78; S, 14.50.

### 3.5. Synthesis of Compounds **4a** and **4b**

Compounds **4a** and **4b** were prepared according to the following general procedure: a mixture of compound **1** (0.244 g, 0.5 mmol), acetyl acetone (0.56 g, 1 mmol), and ammonium acetate (1.0 g) in glacial acetic acid (10 mL) was refluxed for 6 h. The solid product was filtered and recrystallized from DMF/EtOH to afford the desired product in pure form.

#### 3.5.1. 1,1'-(6,6'-(3,4-Diphenylthieno[2,3-*b*]thiophene-2,5-diyl)bis(2-methylpyridine-6,3-diyl))-diethanone (**4a**)

Obtained as a deep yellow powder. Yield 77%; mp: >360 °C. IR (cm^−1^): 1573 (C=N), 1671 (C=O). ^1^H-NMR (DMSO-*d*_6_) δ (ppm): 2.47 (s, 3H, COCH_3_), 2.62 (s, 3H, CH_3_), 7.29–7.59 (m, 5H), 7.95 (d, *J* = 8.0 Hz, 1H, CH pyridine), 8.26 (d, *J* = 8.0 Hz, 1H, CH pyridine). ^13^C-NMR (DMSO-*d*_6_) δ (ppm): 29.4 (CH_3_), 30.6 (COCH_3_), 119.0 (CH), 129.2 (CH), 129.5 (CH), 129.9 (CH), 134.8 (CH), 138.8 (C), 141.8 (C), 147.7 (C), 151.2 (C), 166.1 (C), 194.1 (C, C=O). MS *m*/*z* (70 eV): 560, M^+^ (3%); 522, [M−38]^+^ (25%); 431, [M−1291]^+^ (100%); 326, [M−234]^+^ (94%). Anal. Calcd for C_34_H_26_N_2_O_2_S_2_: C, 73.09; H, 4.69; N, 5.01; S, 11.48; Found: C, 73.20; H, 4.74; N, 4.91; S, 11.40.

#### 3.5.2. Diethyl 6,6'-(3,4-diphenylthieno[2,3-*b*]thiophene-2,5-diyl)bis(2-methylnicotinate) (**4b**)

Obtained as a brown powder; yield: 74%; mp: 245–247 °C. IR (cm^−1^): 1568, 11718. ^1^H-NMR (DMSO-*d*_6_) δ (ppm): 1.29 (t, *J* = 12 Hz, 3H, CH_2_CH_3_), 2.57 (s, 3H, CH_3_), 4.21 (q, *J* = 12.0 Hz, 2H, CH_2_CH_3_), 7.29–7.59 (m, 5H), 8.20 (d, *J* = 8.0 Hz, 1H, CH pyridine), 8.26 (d, *J* = 8.0 Hz, 1H, CH pyridine). ^13^C-NMR (DMSO-*d*_6_) δ (ppm): 29.4 (CH_3_), 30.6 (COCH_3_), 62.17 (CH_2_), 119.1 (CH), 129.2 (CH), 129.6 (CH), 129.9 (CH), 134.8 (CH), 138.8 (C), 141.8 (C), 147.7 (C), 151.2 (C), 166.1 (C), 194.1 (C, C=O). MS data: *m*/*z* (70 eV) 580, M^+^ (4%); 279, [M−301]^+^ (100%); 189, [M−391]^+^ (46%); 104, [M−476]^+^ (33%). Anal. Calcd for C_36_H_30_N_2_O_4_S_2_: C, 69.88; H, 4.89; N, 4.53; S, 10.36; Found: C, 69.89; H, 5.09; N, 4.70; S, 10.17.

### 3.6. Synthesis of 1,1′-(3,4-Diphenylthieno[2,3-b]thiophene-2,5-diyl)bis(4,4,4-triethoxybut-2-en-1-one) *(***5***)*

The title compound was prepared by mixing compound **1** (0.244 g, 0.5 mmol) and triethyl orthoformate (0.148 g, 1 mmol) in presence of ZnCl_2_ (0.5 g) as a catalyst. The mixture was stirred for 4 h followed by addition of DMF/EtOH. The solid product was collected by filtration and recrystallized from DMF/EtOH to afford compound **5** as a brown solid. Yield: 56%; mp: >360 °C. IR (cm^−1^): 1626. ^1^H-NMR (DMSO-*d*_6_): δ (ppm): 1.05 (t, *J* = 12.0 Hz, 3H, CH_3_), 2.95(q, *J* = 12.0 Hz, 2H, CH_2_CH_3_), 5.69 (d, *J* = 12.0 Hz, 1H), 6.53 (d, *J* = 12.0 Hz, 1H), 7.42–7.52 (m, 5H). ^13^C-NMR (DMSO-*d*_6_) δ (ppm): δ, 22.4 (CH_3_), 44.0 (OCH_2_), 127.8 (C) 129.3 (CH), 129.9 (CH), 130.5 (CH), 131.5 (C), 133.3 (C), 136.5 (C), 142.0 (C), 151.0 (C), 182.4 (C=O). MS data: *m*/*z* (70 eV) 694, M^+^ (19%); 480, [M−205]^+^ (32%); 98, [M−587]^+^ (100%). Anal. Calcd for C_38_H_44_O_8_S_2_: C, C, 65.87; H, 6.40; S, 9.26; Found: C, 65.89; H, 6.59; S, 9.40.

### 3.7. Synthesis of 7,7′-(3,4-Diphenylthieno[2,3-b]thiophene-2,5-diyl)di-[1,2,4]triazolo[1,5-a] pyrimidine *(***6***)*

Compound **6** was prepared according to the following procedure: compound **1** (0.244 g, 0.5 mmol) was dissolved in DMF/EtOH (10 mL, 1:3) followed by addition of 3-amino-1*H*-1,2,4-triazole (0.084 g, 1 mmol). The mixture was then refluxed for 6 h and the solid that formed was filtered while hot to afford the desired compound which was recrystallized from DMF/EtOH to afford the title compound in a pure form as pale brown crystals; yield: 68%; mp: 349–351 °C. IR (cm^−1^): 1588. ^1^H-NMR (DMSO-*d*_6_) δ (ppm): 7.41–7.66 (m, 5H), 8.14 (d, *J* = 12.5 Hz, 1H), 8.67 (s, 1H), 8.96 (d, *J* = 12.5 Hz, 1H). ^13^C-NMR (DMSO-*d*_6_) δ (ppm): 115.4 (CH), 125.0 (C), 127.0 (C), 127.3 (CH), 129.1 (CH), 129.9 (CH), 130.2 (C), 132.8 (C), 135.8 (C), 152.8 (C), 154.4 (CH), 159.9 (CH), 162.9 (C). MS data: *m*/*z* (70 eV) 530, M^+^ (1%); 368, [M−162]^+^ (100%); 165, [M−165]^+^ (98%); 105, [M−425]^+^ (40%). Anal. Calcd for C_28_H_16_N_8_S_2_: C, 63.62; H, 3.05; N, 21.20; S, 12.13; Found: C, 63.63; H, 5.31; N, 21.30; S, 12.18.

### 3.8. Synthesis of 4,4′-(3,4-Diphenylthieno[2,3-b]thiophene-2,5diyl)bis(benzo[4,5]imidazo[1,2-a] pyrimidine) *(***7***)*

The title compound was prepared by the following procedure: Compound **1** (0.244 g, 0.5 mmol) was dissolved in ethanol (15 mL) followed by addition of 2-aminobenzimidazole (0.133 g, 1.0 mmol) in DMF/EtOH. The mixture was heated under reflux for 7 h and the solid product was filtered while hot to afford the desired product in a pure form as a white powder. Yield: 70%; mp: 248–350 °C. IR (cm^−1^): 1626. ^1^H-NMR (DMSO-*d*_6_): δ (ppm): 7.42–7.52 (m, 9H), 8.16 (d, *J* = 12.5 Hz, 1H), 8.80 (d, *J* = 12.5 Hz, 1H). ^13^C-NMR (DMSO-*d*_6_) δ (ppm): 112.1 (CH), 115.1 (CH), 122.6 (CH), 125 (C), 127.0 (C), 127.1 (CH), 128.1 (CH), 129.9 (CH), 131.3(C), 136.0 (C), 139.1 (C), 142.0, 148.2 (C), 148.4 (CH), 156 (C). MS data: *m*/*z* (70 eV) 622, M^+^ (25%); 567, [M−45]^+^ (56%); 439, [M−183]^+^ (55%); 183, [M−439]^+^ (100%). Anal. Calcd for C_38_H_22_N_6_S_2_: C, C, 72.82; H, 3.54; N, 13.41; S, 10.23. Found: C, 72.82; H, 3.54; N, 13.41; S, 10.23.

## 4. Conclusions

In summary, we have succeeded in the synthesis of a number of novel substituted thienothiophenes through known chemical routes. The structures of the new compounds were confirmed by means of different spectroscopic methods and by elemental analyses. On the basis of preliminary screening data for these new compounds, the antibacterial and antifungal activity was evaluated. Results revealed that compound **2a** has remarkable activity against both Gram-positive and negative bacteria and against the tested fungi, while compounds **4a**, **5** and **6** showed good activity against Gram-positive bacteria and against fungi. However, because of these promising results of quantitative structure-activity relationship studies, an extensive synthesis of the analogue series of compounds **1**–**7** are under way in order to revise the structural errors of pharmacophoric sites and to carry out more efficient drugs on the basis of a POM better understanding of the relationship between the physicochemical properties and biological activity observed for these compounds.
